# Pliant pathogens: Estimating viral spread when confronted with new vector, host, and environmental conditions

**DOI:** 10.1002/ece3.7178

**Published:** 2021-01-26

**Authors:** Anita Porath‐Krause, Ryan Campbell, Lauren Shoemaker, Andrew Sieben, Alexander T. Strauss, Allison K. Shaw, Eric W. Seabloom, Elizabeth T. Borer

**Affiliations:** ^1^ Department of Ecology, Evolution, and Behavior University of Minnesota St. Paul MN USA; ^2^ Present address: Department of Botany University of Wyoming Laramie WY USA; ^3^ Present address: Odum School of Ecology University of Georgia Athens GA USA

**Keywords:** Barley Yellow Dwarf Virus, novel conditions, pathogen, transmission

## Abstract

1. Pathogen spread rates are determined, in part, by the performance of pathogens under altered environmental conditions and their ability to persist while switching among hosts and vectors.
2. To determine the effects of new conditions (host, vector, and nutrient) on pathogen spread rate, we introduced a vector‐borne viral plant pathogen, Barley Yellow Dwarf Virus PAV (BYDV‐PAV) into hosts, vectors, and host nutrient supplies that it had not encountered for thousands of viral generations. We quantified pathogen prevalence over the course of two serial inoculations under the new conditions. Using individual‐level transmission rates from this experiment, we parameterized a dynamical model of disease spread and projected spread across host populations through a growing season.
3. A change in nutrient conditions (increased supply of phosphorus) reduced viral transmission whereas shifting to a new vector or host species had no effect on infection prevalence. However, the reduction in the new nutrient environment was only temporary; infection prevalence recovered after the second inoculation.
4. *Synthesis*. These results highlight how robust the pathogen, BYDV‐PAV, is to changes in its biotic and abiotic environment. Our study also highlights the need to quantify longitudinal infection information beyond snapshot assessments to project disease risk for pathogens in new environments.

## Graphical abstract

As pathogens move across the world, they encounter new hosts, vectors, and environmental conditions, and their ability to adapt to these new conditions impacts their ability to spread. Here, we demonstrate how a viral pathogen (barley yellow dwarf virus, BYDV‐PAV) was unaffected by switching vector and host species yet there was an initial negative impact of altered host nutrient conditions on virus spread. This effect was reversed after a single serial inoculation demonstrating how pathogens maintain the ability to spread under changing conditions—the core of pathogen spread.

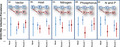

## 1 INTRODUCTION

The spread and impacts of pathogens depend, in part, on their response to altered abiotic or biotic conditions (Auld et al., 2017; Borer et al., 2017; Hall et al., 2009; Keesing et al., 2010; Lloyd‐Smith et al., 2015; Moury et al., 2017; Parrish et al., 2008; Wolfe et al., 2007; Woolhouse & Gowtage‐Sequeria, 2005). For example, many viral pathogens have wide host or vector ranges, which can allow pathogens to spread through heterogeneous environments by taking advantage of different hosts and vector communities (Christian & Willis, 1993; Peck & Lauring, 2018; Pedersen et al., 2005; Power & Flecker, 2015; Varsani et al., 2008). However, generalist and specialist viral pathogens may incur a fitness cost when introduced into alternative hosts (Auld et al., 2017; Elena, 2017; Elena et al., 2009; Mordecai, 2013) or environmental conditions (Lacroix et al., 2014; Smith et al., 2005), suggesting the importance of trade‐offs for long‐term persistence in complex environments. Broadly, understanding how the spread of generalist pathogens is affected by new conditions will improve projections of disease emergence (Becker et al., 2015; Grubaugh et al., 2016).

Abiotic factors, such as climate or host nutrient supplies, have been shown to affect the spread of pathogens (Aylor, 2003; Dunker et al., 2008; Ford et al., 2020; Reinhold et al., 2018; Santini & Ghelardini, 2015; Wyka et al., 2017). For example, human alteration of elemental nutrient conditions is a growing cause of pathogen spread (Hall et al., 2009; Huber et al., 2011; Johnson et al., 2010). Increased use of fertilizers for agricultural purposes and other practices that increase growth‐limiting host nutrients, have fundamentally changed nutrient availability and plant demography (Cadavid et al., 1998; Liu et al., 2010) which can contribute to changes in pathogen dynamics (Walters & Bingham, 2007) by directly altering pathogen replication (Elser et al., 2003; Karpinets et al., 2006) or indirectly by altering the composition or diversity of the host community (Richardson et al., 2002).

Many viral pathogens maintain generality which can proffer greater adaptability in variable environments (Peck & Lauring, 2018), such as the ability to switch hosts (Christian & Willis, 1993) and low host specificity (Pedersen et al., 2005; Varsani et al., 2008). Viral generalists are adapted to multiple host species, maintaining their generality via a diverse viral population and periodically alternating hosts (Elena, 2017). While maintaining generality appears to have low or no fitness cost, virus specialists do incur a fitness cost when introduced into alternative hosts (Elena, 2017; Elena et al., 2009). Infection rates of viral generalists also can initially decrease in a host after switching to a new host (Mordecai, 2013).

Here, we use a model system to determine the effects of changing host and vector species and host nutrient supplies on the spread of a vector‐borne viral plant pathogen. We maintained a generalist viral pathogen, Barley Yellow Dwarf Virus serotype PAV (BYDV‐PAV), in a single host and vector species under constant nutrient conditions for several thousand (estimated 1,800 to 6,000) viral generations. We subsequently performed two rounds of serial inoculations in both the source conditions, and then introduced it into a new (i.e., not encountered recently by the pathogen) host and vector species and under new nutrient conditions. Using this experiment, we address the following hypotheses:

Hypothesis 1 *A generalist pathogen introduced into a new environment will initially experience slower spread rate, measured as reduced infection prevalence*. We predict BYDV‐PAV infection prevalence will be reduced compared to natal conditions when introduced into new vector, host, and nutrient conditions due to the cost of maintaining generality (Auld et al., 2017; Mordecai, 2013).

Hypothesis 2 *The effects on pathogen spread after introducing a generalist virus into new conditions will weaken after serial inoculations*. We predict initial reduction in BYDV‐PAV spread rate under new conditions will weaken with time as the virus is maintained under the new conditions due to local adaptation facilitated by viral replication (Elena, 2017).

To address these two hypotheses, we quantify BYDV‐PAV prevalence under both source conditions and under new vector, host, and nutrient conditions for two serial inoculations. Then, we quantify transmission rate at the level of an individual host. Using these rates, we parameterize a dynamical model of disease spread, projecting how changes in transmission at the individual host level manifest in spread rates across host populations through a growing season.

## 2 METHODS

### 2.1 Study system

#### 2.1.1 Barley yellow dwarf virus

The focal pathogen, BYDV‐PAV serotype (*Luteoviridae*), is member of the barley and cereal yellow dwarf virus (B/CYDV) group which is known to infect at least 150 grasses including wild grasses and crops such as wheat, barley, and oats (D'Arcy & Burnett, 1995; Irwin & Thresh, 1990). This generalist virus was originally described by Rochow (1970) and is characterized as a New York BYDV‐PAV isolate. The isolate was maintained by Dr. Stewart Gray at Cornell University and then at the University of Minnesota since 2013 under constant host, nutrient, and vector conditions (see Section 2.2.1 and Appendix S1). The virus is a positive, single‐stranded RNA virus that is considered to be phloem‐limited (Miller & Rasochová, 2002). BYDV‐PAV is transmitted obligately by aphid vectors in a persistent, circulative, and nonpropagative manner (Gray & Gildow, 2003; Miller & Rasochová, 2002).

#### 2.1.2 Vector and host material

Two aphid species, *Rhopalosiphum padi* and *Sitobion avenae,* are effective vectors of BYDV‐PAV, a widespread BYDV strain. Aphids are phloem‐feeding insects (Aphidae) that feed on a wide variety of annual and perennial plants. At least 25 aphid species can efficiently transmit B/CYDVs, though their competence (i.e., the capability of the aphid to transmit a particular virus) as vectors varies widely among viruses (D'Arcy & Burnett, 1995). The molecular mechanism behind the B/CYDV‐vector specificity (i.e., the relative transmission ability of different virus isolates by different aphid species) involves a ligand/receptor interaction between the virus and vector at the aphid's accessory salivary gland (Gildow & Gray, 1993; Reavy & Mayo, 2002).

*Rhopalosiphum padi* generally has a higher average acquisition rate of BYDV‐PAV than *S. avenae*, which is thought to be associated with differences in vector feeding behavior (Gray et al., 1991). However, even within a single aphid species, B/CYDV‐vector specificity differs based on aphid biotypes, or clones, making efficiency difficult to estimate across different infection events (D'Arcy & Burnett, 1995).

Conditions that impact vector specificity, the relative transmission ability of different virus isolates by different aphid species, or vector competence, the capability of an organism to transmit a particular pathogen, also affect pathogen spread. For example, a broad vector range can allow pathogens to spread more widely than pathogen with specialist vector (Lacharme‐Lora et al., 2009). However, if a virus switches to a vector with lower specificity, transmission to the host may decrease (Rochow et al., 1975).

Variation among host traits (Cronin et al., 2010; Malmstrom et al., 2005) and among host genotype (species varieties or lines) (Chain et al., 2007), whether naturally occurring or through selective breeding, can affect resistance and tolerance of B/CYDV infection (Chain et al., 2005; D'Arcy & Burnett, 1995; Jin et al., 1998; Koev et al., 1998; Schreurer et al., 2001; Scholz et al., 2009). In this study, we examine transmission in two grass (Poaceae) hosts, *Hordeum vulgare* (domestic barley) and *Avena sativa* (domestic oats). These hosts belong to the same monophyletic group but diverged 25 million years ago (Gaut, 2002). *H. vulgare* and *A. sativa* were chosen as hosts in this study because BYDV‐PAV has been shown to maintain infectivity after switching from oat to barley hosts (Chain et al., 2007). Both species are grown as food grade cereal crops and can be infected by and show symptoms of BYDV‐PAV. *H*. *vulgare* seed was reared from a disease‐resistant line while *A. sativa* was reared from a disease susceptible line (see Host Source Conditions in Appendix S1). Under similar laboratory conditions, we have observed that these two species have similar germination timing and plant growth so that when aphids were added, growth stages and plant development were relatively equal.

#### 2.1.3 Effects of nutrients

BYDV dynamics can be affected by the supply rates of host nutrients including phosphorus and nitrogen (Lacroix et al., 2017). Nitrogen can either directly increase or decrease a plant's defenses to a pathogen. The addition of NO_3_‐ increases the production of established defense signals, and nitric oxide (NO) can reduce a pathogen's ability to penetrate a plant by inducing stomatal closure; however, ammonium (NH_4_) can compromise defense by increasing amino acid and sugar production inside the plant, increasing the nutrients available to the pathogen (Mur et al., 2017). Nitrogen addition has also been shown to increase virus transmission by increasing vector fecundity through improved nutrition (Borer, Adams, et al., 2009; Borer, Mitchell, et al., 2009; Strauss et al., 2020). Increased infection prevalence in the presence of added phosphorus is likely due to higher viral titer in the host enhancing transmission (Borer et al., 2010). Generally, viral replication rates increase inside the host with the addition of phosphorus, possibly because phosphorus or phosphorus‐demanding organelles such as ribosomes limit viral reproduction (Clasen & Elser, 2007); however, viral replication rates also can decrease when the addition of phosphorus increases the host's immune response so the host can actively suppress replication (Mandadi & Scholthof, 2013).

### 2.2 Experimental design

#### 2.2.1 Natal conditions

Our experiment used BYDV‐PAV viral cultures that had been maintained in the laboratory (see Viral Culture Source in Appendix S1) using a single aphid species, *S*. *avenae*, on a single host species, *A*. *sativa*, under low nutrient conditions for 251 days (see detailed information regarding vector colony and host plant source in Appendix S1, Vector Conditions and Host Conditions). Aphid population growth under these natal conditions produces approximately 12 generations of *S. avenae* (Dedryver et al., 1998), and approximately 1,800 to 6,000 generations of BYDV‐PAV (Yarwood, 1956).

#### 2.2.2 Treatment conditions

**FIGURE 1:**
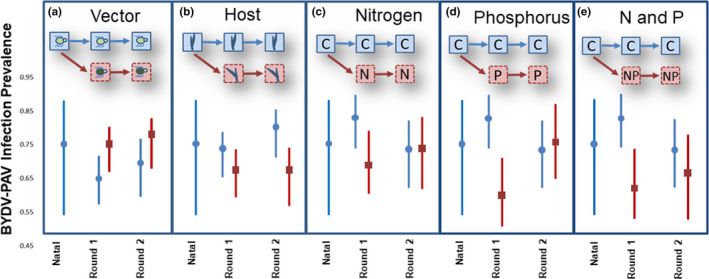
Altered nutrient conditions cause a reduction in BYDV‐PAV infection prevalence. In each panel, infection prevalence for Round 1 and Round 2 for the respective treatment, averaged across all other treatments. For (a) vectors *S. avenae* (blue line) and *R. padi* (red line), (b) hosts *A. sativa* (blue line) or *H. vulgare* (red line), (c) control (blue line) or nitrogen addition (red line), (d) control (blue line) or phosphorus addition (red line), (e) control (blue line) or nitrogen and phosphorus (N and P) (red line) nutrient treatments. The blue line at the left of each panel represents the natal infection prevalence which was calculated using the average prevalence over all treatments. Error bars represent 95% confidence intervals

We experimentally exposed natal viral cultures to a range of conditions consisting of two aphid vectors, two plant hosts, and four nutrient conditions for a total of 16 treatments. The treatments consisted of a full cross of vector (two conditions), host (two conditions), and nutrient (four conditions) across the natal, *S. avenae*, or new aphid vector, *R. padi*, the natal, *A. sativa*, or new host species, *H. vulgare,* and the nutrient conditions. The nutrient conditions included nitrogen (NH_4_NO_3_), phosphorus (KH_2_PO_4_), nitrogen plus phosphorus, or no additional nutrients. Each treatment was repeated eight times over three consecutive temporal blocks for a total 24 replicates per treatment. For each block of the experiment, 70 seeds from each host plant species were planted for a total of 140 plants. Each block had eight replicates per treatment with 12 additional seeds planted to account for the possibility of for failed germinations. The plants were watered with the four nutrient treatments: nanopure water only (Control), 10% nitrogen solution, 10% phosphorus solution, or 10% nitrogen & phosphorus solution all based on a Half‐Strength Hoagland's solution (Hoagland & Arnon, 1950) which are consistent with previous experiments (Lacroix et al., 2014). The first inoculation that introduced the virus to new biotic and abiotic conditions will be referred to as Round 1. We then performed a second inoculation (referred to as Round 2) in vivo such that all treatments were applied to a set of hosts that maintained the treatment where plant tissue from Round 1 treatments was used to infect the aphids used in Round 2. A simple schematic depicting the inoculation and treatment conditions is represented in Figure 1. Assessing viral evolution and population dynamics after serial passages is not uncommon (Bartels et al., 2016; Kurath & Palukaitis, 1990; Schneider & Roossinck, 2000; Sylvester et al., 1974) but quantifying virus transmission in serially passaged viruses after switching abiotic or biotic conditions has rarely been performed. All plant tissues were collected between June 5th, 2017, and October 10, 2017. All plant tissue was collected and preserved at −20°C for molecular processing.

### 2.3 Virus inoculation

Each block as described under *Treatment Conditions*, included two inoculation rounds. During the first inoculation (Round 1) of the experiment, 360 live, adult‐sized aphids of both *R. padi* and *S. avenae* were removed from uninfected plants and transferred to 25 ml cork sealed tubes (24×) each containing 30 aphids of the same species. Leaf tissue from approximately four‐week‐old plants confirmed to be infected with BYDV‐PAV was clipped, and 4–6 cm of infected tissue was transferred into each tube containing nonviruliferous aphids. Aphids remained in cork sealed tubes for 48 hr such that they became viruliferous from feeding on infected plant tissue, meaning the aphids were then able to transmit the virus. After 48 hr, aphids were moved to uninfected plants for the initial inoculation period. Plants used for the initial inoculation were uninfected prior to aphid exposure as the plants remained isolated from aphids and other insects and there is no evidence of vertical transmission of BYDV‐PAV in hosts.

We controlled for factors known to influence transmission efficiency including length of feeding period on infected tissue and age of host tissue (Gray et al., 1991). To do this, a single 2.5 × 8.5 cm, 118 μm polyester mesh cage was attached to the oldest leaf on each 17‐day‐old experimental plant. Five viruliferous aphids were transferred into each polyester mesh cage which was then sealed. The experimental plants containing the caged aphids were then placed in a growth chamber and aphids fed for approximately 96 hr, after which the aphids were killed to end transmission.

At the start of the second inoculation (Round 2), the experimental plants from Round 1 were destructively harvested and the polyester mesh cages were removed. Each 8.5‐cm leaf enclosed by the cage was cut from the plant and transferred to a clean 25‐ml tube while the aboveground plant tissue was stored at −20°C. Once all tissues were collected at the end of the experiment, BYDV‐PAV infection status (presence/absence) was assessed using polymerase chain reaction (see Virus detection in Appendix S1). Ten aviruliferous aphids (i.e., not yet carrying a virus) of each species were collected from vector source conditions, transferred into each of the tubes respective to the treatment, and allowed to feed for 48 hr. Five aphids from each tube were transferred to a new experimental plant raised under the treatment conditions and cage‐sealed; the remaining five aphids were discarded. During Round 2, the treatments with *R*. *padi* in the sixth block only contained one aphid per cage due to *R*. *padi* colony depletion. All other treatments contained five aphids per cage per block. The experimental plants were subjected to feeding period of 96 hr after which the aphids were killed. BYDV‐PAV infection status (presence/absence) was assessed using polymerase chain reaction (see Virus detection in Appendix S1).

### 2.4 Statistical analyses

To quantify prevalence (i.e., proportion of infected plants) for each treatment, we divided the number of plants with confirmed BYDV‐PAV infection by the total number of plants in the treatment. To determine whether treatment affected the probability of infection, we fit a logistic regression model in R version 3.5.1 (R Core Team, 2013) using the glmmTMB library (Borer et al., 2017) with BYDV‐PAV infection status of each experimental plant (presence/absence) as a binomial response variable. Temporal block was included as a random effect (intercept; 6 levels, with 3 blocks each for the first and second infection rounds). Visualization of prevalence in the different treatments (Figure 1) suggested that two‐way and even three‐way interactions among vector, host, N, P, and round may have influenced the probability of infection. However, a model with all three‐way interactions produced 26 terms and proved difficult to interpret. Therefore, we used AIC to determine which two and three‐way interactions improved model fit (Sakamota et al., 1986). We used the dredge function in the MuMIn library (Barton, 2020) to consider all models with main effects of vector, host, N, P, and round, and up to all three‐way interactions (6,212 total models). The top ten models (ten lowest AIC scores) were inspected to identify the best “consensus” model (Table S3). We included terms in the consensus model if they were present in all ten (7×) or at least eight (3×) of the top ten models. We excluded terms if they were not present (NA) in any (7×), or two or fewer of the top ten models (2×). The host: vector interaction was present in five of the top ten models, and we conservatively included it in our consensus model. The final model contained the following: infection ~ (host * N * P) + (round * N * P) + (host * round * P) + (P * vector) + (host * vector) + (1 | block).

### 2.5 Dynamical model and virus transmission projections

To compare changing natal vector, host, or nutrient conditions on disease spread throughout an entire host population, we simulated a model of vectored‐pathogen spread. To estimate realized BYDV‐PAV transmission rates from experimental results, we simulated a simplified susceptible‐infected (SI) model to our data,

(1) $$\frac{dS}{dt}=‐SA\beta_{1}\;\frac{dI}{dt}=SA\beta_{1},$$

where *S* is the density of susceptible (healthy) plants in the experiment (plants/pot), *I* is the density of infected plants (units: plants/pot), *A* is the number of infectious aphids per plant (aphids/plant), and *β*_1_ is a per‐aphid transmission coefficient (plants aphid^−1^ day^−1^). *β_1_* is the rate at which an individual aphid transmits the virus to a susceptible plant. Broadly, this approach allowed us to quantify how different treatments caused variation in this important parameter *β*_1_ (listed with confidence intervals in Table S1). Solving the differential equation for the final density of susceptible plants *S_f_* after *t* days yields

(2) $$S_{f}=S_{i}\exp\left(‐A\beta_{1}t\right),$$

where *S_i_* is the initial density of susceptible plants. We estimated transmission coefficients (*β*_1_'s) by fitting this equation to different treatments of our experimental data using maximum likelihood and the bbmle package in R (Bolker, 2008). We treated each experimental plant as an independent observation that began as an uninfected host (*S_i_* = 1) and either remained uninfected or became infected (*S_f_* = 0 or 1, respectively) during the time the aphid was allowed to feed (*t* = 4 days). The binomial distribution served as our likelihood function. This approach allowed us to estimate comparable transmission coefficients (units: plants aphid^−1^ day^−1^), even though some plants in the experiment were exposed to different numbers of aphids. Specifically, five infectious aphids (*A* = 5) were added to most plants, but we were limited by *R. padi* population size for the final round of the experiment. Therefore, each plant in the *R. padi* treatments in the final round of the experiment received only a single aphid (*A* = 1), which we account for in the above parameter estimation. We resampled data 10,000 times within each treatment to bootstrap 95% confidence intervals around each transmission coefficient.

We fit separate models to estimate transmission coefficients across treatments, comparing changing host, changing vector, or changing phosphorus condition (since nitrogen was found to be nonsignificant) both initially (Round 1) and after the serial inoculation (Round 2) which potentially contains double the number of viral generations (estimated at 528–1,760 viral generations) as Round 1. The data were subdivided by treatment and round (averaged across other factors) to determine the effect of changing hosts, changing vectors, and changing phosphorus amounts, respectively. Nitrogen was not included in the third contrast as the logistic regression model showed nitrogen had no additive or interactive effect on the probability of infection (Table 1).

**TABLE 1:** Probability of infection as a function of vector, host, and nutrient addition using logistic regression model

Response	Fixed effects	Estimate	SE	*z* value	Pr(>|*z*|)
Host infection status	(Intercept)	2.360	2.244	1.052	0.293
	Host *H.vulgare*	1.709	1.148	1.489	0.137
	Nutrient N	−1.735	1.053	−1.648	0.099
	Nutrient P	−2.160	1.275	−1.695	0.090
	Round	0.526	1.423	0.372	0.710
	Vector *S. avenae*	−1.471	0.415	−3.546	**0.000**
	Host *H. vulgare*: N	−1.012	0.660	−1.534	0.125
	Host *H. vulgare*: P	−3.283	1.479	−2.221	**0.026**
	Nutrient N: P	1.752	1.442	1.215	0.224
	Nutrient N: Round	1.246	0.682	1.826	0.068
	Nutrient P: Round	0.716	0.824	0.869	0.385
	Host H. vulgare: Round	−1.908	0.688	−2.772	**0.006**
	Nutrient P: Vector *S. avenae*	0.869	0.456	1.906	0.057
	Host *H. vulgare*: Vector *S. avenae*	0.639	0.455	1.403	0.161
	Host *H. vulgare*: N: P	2.781	0.917	3.032	**0.002**
	Nutrient N: P: Round	−2.010	0.956	−2.102	**0.036**
	Host *H. vulgare*: P: Round	2.162	0.965	2.239	**0.025**

Bold font indicates statistical significance.

Using the above parameterized transmission coefficients, we examined how new vector, host, or nutrient conditions affect BYDV‐PAV, scaling our transmission results for individual hosts to estimate disease spread across a growing season for an entire host population. To do so, we created a model of vectored‐pathogen spread by modifying the classic susceptible‐infected framework to model dynamics of healthy and infected plant hosts (*P_h_* and *P_i_* respectively) as well as nonviruliferous and viruliferous vectors (*V_h_* and *V_i_*, respectively) using a system of ordinary differential equations (Keeling & Rohani, 2008; Shaw et al., 2017). Dynamics of host and vector populations occur such that:

(3) $$\frac{dP_{h}}{dt}=\beta_{1}V_{i}\frac{P_{h}}{T},$$

(4) $$\frac{dP_{i}}{dt}=\beta_{1}V_{i}\frac{P_{h}}{T},$$

(5) $$\frac{dV_{h}}{dt}=r_{h}\left(V_{h}+V_{i}\right)\frac{P_{h}}{T}\left(1‐\frac{\left(V_{h}+V_{i}\right)\frac{P_{h}}{T}}{KP_{h}}\right)‐\beta_{2}V_{h}\frac{P_{i}}{T}‐\mu \alpha V_{h},$$

(6) $$\frac{dV_{i}}{dt}=r_{i}\left(V_{h}+V_{i}\right)\frac{P_{i}}{T}\left(1‐\frac{\left(V_{h}+V_{i}\right)\frac{P_{i}}{T}}{KP_{i}}\right)+\beta_{2}V_{h}\frac{P_{i}}{T}‐\mu \alpha V_{i}.$$

Here, *T* is the total number of hosts in the community, *β*_1_ is the transmission coefficient from vector to host species as derived from the experiment (Equations 1 and 2), *β*_2_ is the transmission coefficient from host species to vector, *r_h_* and *r_i_* are the intrinsic growth rates of vectors on healthy and infected hosts, respectively, *K* is the carrying capacity of vectors on a single host, *α* is the vector departure rate from hosts, and *μ* is the dispersal‐induced vector mortality rate (see Appendix S1, Figure S1 legend for model parameterization). Transmission in our model is density‐dependent for both the hosts and vectors. We include multiple generations of aphids within a single growing season where aphid population dynamics follow logistic growth. We assume that vectors born on infected plants become infected (Shaw et al., 2017) and that vector intrinsic growth rates depend on the host infection status, matching previous experimental observations (Jiménez‐Martínez et al., 2004). We additionally account for aphid mortality due to dispersal, as mortality is estimated to be as high as 99.4% during dispersal (Ward et al., 1998), and thus, this loss may significantly alter vector and disease dynamics. A sensitivity analysis was performed to show how model dynamics vary with parameters and the relative importance of transmission from vectors to host (our focus here) versus other model parameters (Appendix S1, Figure S2, Table S4).

Using the above model, we compared the percent of the host population that is infected through time with different transmission rates for our experimental treatments. All model parameters were kept constant, except transmission from vector to plant host (*β*_1_), which was varied according to experimental results. We examined dynamics in a host population consisting of 1,000 individual hosts (*T*). We initialized the model with 1% of the host population infected at the beginning of the season and 50 nonviruliferous aphids initially at time *t* = 0. We compared disease dynamics from Round 1 and Round 2 between the natal and new conditions, where we varied (a) the vector species, (b) the host species, and (c) nutrient conditions. Transmission rates ranged from 0.048 to 0.090 plants aphid^−1^ day^−1^ (Table S1). The model was simulated in R version 3.5.1 (R Core Team, 2013) using the deSolve library for solving systems of differential equations (Bates et al., 2014).

## 3 RESULTS

### 3.1 Experimental results: Do new conditions change pathogen spread?

Infection prevalence was lower in the natal vector, *S. avenae* (*p* = 0.000) than in the new vector, *R. padi*. The proportion of successful viral transmission events by *S. avenae* (prevalence = 0.656, 95% CI = 0.584, 0.721) was lower than *R. padi* (prevalence = 0.747, 95% CI = 0.678, 0.806 (Figure 1, panel a) (Table S2). BYDV‐PAV infection prevalence was affected after being introduced into the new host. There was a significant interaction between *H. vulgare* and round (*p* = 0.006) which appears to be in large part, due to the consistently low infection prevalence in *H. vulgare* during Round 2 (Figure 1, panel b).

Introducing BYDV‐PAV into hosts growing with elevated phosphorus initially decreased BYDV‐PAV prevalence (prevalence = 0.618, 95% CI = 0. 514, 0.712) relative to the control level of nutrients (prevalence = 0. 835, 95% CI = 0. 745, 0. 899) (Figure 1, panel d) (Table S2). The addition of phosphorus reduced the probability of BYDV‐PAV infection in *H. vulgare* (*p* = 0.026) but did not appear to reduce the probability of infection in *A. sativa* (Table 1). However, the reduced infection in *H. vulgare* under elevated P was transitory and disappeared in the second round of inoculations (*p* = 0.025).

Nitrogen, alone, did not impact the probability of BYDV‐PAV infection (*p* = 0.099, Table 1) (prevalence = 0.710, 95% CI = 0.610, 0.793) (Figure 1, panel c); however, when applied in combination with phosphorus, nitrogen could have opposing effects (prevalence = 0.643, 95% CI = 0.536, 0.737) (Figure 1, panel e). When both host species were fertilized with nitrogen and phosphorus, the probability of infection was lower after the switch and continued to have a lower probability of infection (*p* = 0.036, Table 1) (Figure 1, panel e). However, *H. vulgare* appeared more susceptible to BYDV‐PAV infection when treated with nitrogen and phosphorus because the probability of infection was higher when both nutrients were added to this host (*p* = 0.002).

### 3.2 Dynamical modeling results: Can slowed pathogen spread recover after repeated transmission events?

The dynamical model projected that the time to reach 50% infection (i.e., half of all hosts infected; *P_i_*/(*P_h_* + *P_i_*) = 0.5) was similar between rounds for both vector and host treatments. Time to reach 50% infection was faster for both vectors using Round 2 parameters (*S. avenae* = 66 days (62–70 CI); *R. padi* = 59 (54–63) days) compared with Round 1 (*S. avenae* = 68 days (65–71); *R. padi* = 63 days (60–66)). The virus reached 50% infection faster when transmitted by *R. padi* than *S. avenae* (Appendix S1, Figure S1A,B). Similarly, the virus reached 50% infection in less time when infecting the host *A. sativa* than *H. vulgare* in both rounds. The time to reach 50% infection was faster in both hosts in Round 2 relative to Round 1 (*A. sativa* = 64 (61–67) and 58 (54–62) days; *H. vulgare* = 67 (63–71) and 66 (63–71) days for Round 1 and Round 2, respectively) (Appendix S1, Figure S1C,D).

**FIGURE 2:**
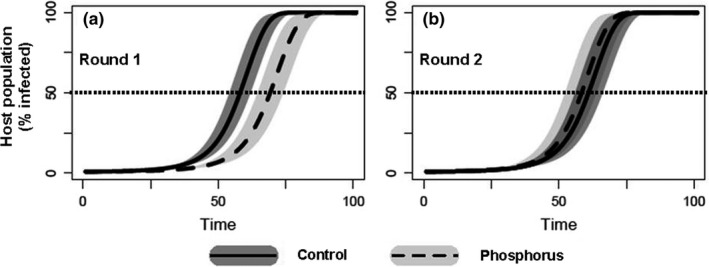
Addition of phosphorus showed contrasting effects of pathogen spread. Percent of infected hosts as modeled for a single growing season when data are modeled for Round 1 (panel a), only, or modeled for Round 2 (panel b) for the control nutrient conditions (solid line) and increased phosphorous (dashed lines). Projections estimate Round 1 time to reach 50% BYDV‐PAV infection for control is 59 days and phosphorus is 70 days. However, the relative relationship reversed between Round 1 and Round 2 as projections estimate that Round 2 phosphorus addition treatment reaches 50% infection in 59 days and the control nutrient treatment reaches 50% infection in 61 days. Shaded regions show 95% confidence intervals, estimated from the bootstrapped 95% confidence intervals of vector to plant transmission coefficients (β_1). Model parameter details including model equations in Appendix S1, Figure S11 and Eq. A1 through Eq. A6

The addition of phosphorus showed contrasting effects of BYDV‐PAV spread (Figure 2, panel a and b). Phosphorous addition slowed BYDV‐PAV transmission with parameters estimated from Round 1, (phosphorus = 70 (65–75) days; control = 59 (54–63) days); however, this effect disappeared in Round 2 transmission parameterizations (phosphorus = 59 (52–65) days; control = 61 (56–67) days).

## 4 DISCUSSION

In this study, we used a model viral pathogen to determine how new biotic and nutrient conditions alter transmission and the spread of a vector‐borne viral plant pathogen. Whereas introducing BYDV‐PAV into a new vector or host species did not initially affect infection prevalence, we found that prevalence significantly decreased when BYDV‐PAV was introduced into a new environment with elevated phosphorus supply. However, our second serial inoculation revealed that this initial observation of reduced infection prevalence is not an accurate predictor of longer‐term BYDV‐PAV spread, but rather a transient response to new conditions. Comparing the first inoculation round to the second inoculation round elucidated how the reduction in infection prevalence was transitory and recovered quickly, even with continued elevated phosphorus conditions.

We found that the viral pathogen, BYDV‐PAV, had higher infection prevalence in the new vector than the natal vector, suggesting the higher specificity of the new vector overrode any cost of novelty. While changes in biotic conditions have been shown to alter the degree of vector competence (Lopes et al., 2009), our study controlled for factors shown to contribute to variation in BYDV‐PAV transmission efficiency (e.g., vector acquisition period, nutrient conditions, and host preference) (Gray et al., 1991). Thus, the results of this work demonstrate that inherent vector competence is more important for BYDV‐PAV spread than the natal vector species. While vector specificity affects BYDV‐PAV spread, both vectors retained BYDV‐PAV competence even after thousands of viral generations suggesting BYDV‐PAV is unlikely to experience reduced vector transmission even in vectors that are rarely encountered. Maintaining vector generality is important because there are at least 25 species of aphids known to transmit at least one B/CYDV (D'Arcy & Burnett, 1995), and our results suggest that generalism can be conserved, even over thousands of viral generations with transmission from only a single vector species. Vector competence can be as simple as a few genetic differences between viral strains, implying that a few mutations could potentially open the door for many more competent vectors. The ability of a pathogen to increase transmission by expanding into different competent vectors has clear implications for a wide range of fields including those related to human, wildlife, livestock, and crop health.

As predicted, we found lower BYDV‐PAV infection prevalence in the new host, *H*. *vulgare*. This difference could reflect differential growth of the virus within the host during the initial introduction since the successful transmission in the second inoculation required the aphid to acquire the virus from the previous host. Furthermore, the virus may be suppressed by a disease‐resistant host germline (Holmfeldt et al., 2007; Middelboe et al., 2009; Rohrmann, 2013). For example, in this study, *H*. *vulgare* may have experienced consistent lower transmission across infection rounds because the seeds were acquired from a disease‐resistant germline containing the B/CYDV resistant Yd2 gene—a gene shown to increase infection tolerance and reduce disease symptoms in barley (Rasmusson & Schaller, 1959).

In contrast with new vectors and hosts, virus transmission declined when initially exposed to elevated phosphorus conditions. However, this negative effect was transient, disappearing in the second round of serial inoculations. In addition, the dynamic model projections showed no noticeable effect on spread through a host population once the transient decrease in transmission disappears; the addition of phosphorus did not change the likelihood of reaching 50% infection during the growing season after the second inoculation. The initial negative effect of increased phosphorus aligns with other experiments that have demonstrated a negative effect of phosphorus on BYDV‐PAV prevalence in growth chamber experiments (Lacroix et al., 2014).

Host nutrition can directly or indirectly alter pathogen replication (Huber et al., 2011; Johnson et al., 2010), suggesting that a host plant's ability to resist infection may explain the reduced BYDV‐PAV prevalence under elevated nutrients. Most plants have evolved complicated antiviral defenses (Mandadi & Scholthof, 2013), and higher nutrients can increase a host's disease resistance to infection (Dordas, 2008). However, the subsequent inoculation suggested that, if this mechanism is operating here, BYDV‐PAV was eventually able to overcome host defenses under the elevated phosphorus treatment, leading to substantially increased infection prevalence in the second round. Additionally, the observed lack of significant change in response to increased nitrogen may have resulted from fertilization with two nitrogen types, NO_3_‐ and NH_4_, which have opposing effects on plant immunity; their interaction could have produced a null response. In particular, nitrate (NO_3_‐) has been shown to increase defense signals, whereas ammonium (NH_4_) can compromise defense (Mur et al., 2017). These results suggest the importance of context dependence: If nutrients act on host resistance, the specific nutrient combination in the new environment will determine the initial spread of the virus.

We predict that while the virus may not spread as quickly in the new host species as the natal species, BYDV‐PAV would maintain host generality and continue to spread. Although hosts spanning these genera co‐occur regularly in grasslands, our focal hosts diverged 25 million years ago (Gaut, 2002), reemphasizing the capacity of the generalist virus to infect and spread across divergent phylogenetic clades. Human manipulation of land use for urban and agricultural needs can introduce non‐native plant hosts that would not typically co‐occur in an area (Seabloom et al., 2006), creating more potential for BYDV‐PAV to encounter novel, distantly related host species.

While generalist pathogens are capable of infecting multiple host species, we should not expect equal spread across host species. In this study, both hosts were BYDV‐PAV susceptible and both vectors were BYDV‐PAV competent, as expected, yet infection prevalence experiments show that in each case, one vector or host species was always more likely to transmit the virus and become infected. This result suggests that host and vector competence is more of an innate characteristic and is not easily changed, which may explain the large host and vector range of many viruses (Power & Flecker, 2015). In more complex field settings, host and vector community interactions may also be driven by vector foraging behavior. For example, aphids have preference for different host species (Borer, Adams, et al., 2009; Borer, Mitchell, et al., 2009), and preferred hosts have higher disease prevalence in field settings (Seabloom et al., 2013).

As human activities are increasing the exposure of pathogens to different hosts, vectors, and shifting environmental conditions, understanding the risk posed by increasing pathogen spread depends on effective predictions of the pathogen's performance under these new conditions. Our results demonstrate that the viral pathogen, BYDV‐PAV, does not readily lose vector or host competence. This case study illustrates how viral pathogens can maintain large host and vector ranges, even after thousands of generations in a uniform environment. The capacity to persist in changing environments, hosts, and vectors is among the reasons vector‐borne viruses are such globally important pathogens.

## CONFLICT OF INTEREST

We are unaware of any conflicts of interest associated with this publication.

## AUTHOR CONTRIBUTION

**Anita Porath‐Krause:** Conceptualization (equal); Data curation (supporting); Formal analysis (supporting); Methodology (equal); Project administration (lead); Writing‐original draft (lead); Writing‐review & editing (lead). **Ryan Campbell:** Conceptualization (supporting); Data curation (lead). **Lauren Shoemaker:** Formal analysis (equal); Writing‐original draft (supporting); Writing‐review & editing (supporting). **Andrew Sieben:** Data curation (equal); Writing‐review & editing (supporting). **Alex Strauss:** Formal analysis (equal); Writing‐original draft (supporting); Writing‐review & editing (supporting). **Allison Shaw:** Formal analysis (supporting); Writing‐review & editing (supporting). **Eric Seabloom:** Conceptualization (equal); Funding acquisition (equal); Investigation (equal); Methodology (equal); Project administration (supporting); Resources (equal); Supervision (equal); Writing‐original draft (supporting); Writing‐review & editing (supporting). **Elizabeth Borer:** Conceptualization (equal); Funding acquisition (equal); Investigation (equal); Methodology (equal); Project administration (supporting); Resources (equal); Supervision (equal); Writing‐original draft (supporting); Writing‐review & editing (supporting).

## Supporting information

Appendix S1

## Data Availability

Infection data from this study are available from the Dryad Digital Repository: https://doi.org/10.5061/dryad.6djh9w10j.
