# Supplementary material for: Pliant pathogens: Estimating viral spread when confronted with new vector, host, and environmental conditions
Source: Ecol Evol. 2021 Jan 26;11(4):1877–87. doi: 10.1002/ece3.7178 (PMC7882977; doi:10.1002/ece3.7178)
Supplement: Supplementary file 1 — Appendix S1 [file ECE3-11-1877-s001.docx]

**Supplementary Material: Appendix 1.**

“**Pliant pathogens: Estimating viral spread when confronted with new vector, host, and environmental conditions**”

**APPENDIX 1**

Here, we present supplementary methods, tables, and figures. In the supplementary methods, we first detail the source and experimental conditions of the viral culture, vectors, and hosts used in the study. Specifics of BYDV-PAV virus detection are included in the subsequent section. We also describe the methods for a sensitivity analysis to examine the relative importance of the observed variation in transmission from plants to vectors ($\beta_{1}$) relative to the other demographic and transmission parameters. Supplementary material Table A1, lists the transmission coefficients ($\boldsymbol{\beta}_{\boldsymbol{1}})$ and confidence intervals used to fit the experimental data in the parameterized model. Supplementary Table B1 reports the BYDV infection prevalence with confidence intervals and margin of error for each of the 16 treatments. The logistic regression model selection is detailed in Supplementary Table C1. Supplementary material Table D1 lists the parameters, description, and mean and range values for the disease transmission model (eq. 3 – eq. 6). Supplementary material Appendix 1, Fig. A1 shows the projected time for the vector or host to reach 50% infection were similar for both inoculation series according to the dynamical model. Supplementary material Appendix 1, Fig. B1 shows the effect size of variation in model parameters on the number of days it takes to reach 50% infection of the host population.

Supplementary Methods

**Viral culture source**

Initial virus colonies were started with BYDV-PAV infected *A. sativa* tissue provided from Dr. Stewart Gray at Cornell University in 2013. Aphids were allowed to feed on the infected tissue for an approximately 48-hour acquisition period.  The viruliferous aphids were then transferred to uninfected *A. sativa* plants and allowed to feed for an approximately three-week inoculation period in a growth chamber under natal conditions.  Aphids were subsequently killed and the newly BYDV-PAV infected *A. sativa* plants were placed in a different, aphid-free growth chamber, to prevent any chance of unintentional transmission. Tissue from infected, aphid-free plants was used for each acquisition.  Virus presence was routinely checked using BYDV-PAV specific PCR-based methods (see Virus detection).

**Vector source conditions**

The vectors *Rhopalosiphum padi* and *Sitobion avenae* were obtained from Dr. George Heimpel at the University of Minnesota.  Disease-free (hereinafter referred to as non-viruliferous) colonies of both aphid species were maintained in lab on single potted *Avena sativa* Coast Black Oats for at least 10 days.  After approximately two weeks of population growth, 25-30 apterous (non-winged) aphids of each species were transferred to empty 25 ml glass tubes. Alates (winged aphids) were regularly discarded as they move greater distances than their apterous counterpart (Irwin & Thresh 1988) and we wanted to minimize risk of cross-contaminating colonies.  To begin new colonies, a 4-8 cm piece of healthy *A. sativa* tissue was placed in the vial. Aphids were given a two-hour period to adhere to the leaf before transferring the aphids and plant tissue into a new, uninfected pot of *A. sativa* to allow for continuous colony growth. Non-viruliferous aphids feeding on *A. sativa* were kept in growth chambers for approximately three weeks until next feeding transfer.

**Host source conditions**

Two host plant species, *Avena sativa* (received August 2015, *Avena sativa* L. cv Coast Black Oat National plant germplasm system, USDA; USA) and *Hordeum vulgare* (received January 2016, Organic Quest Barley, Albert Lea Seed House 1414 West Main St, Albert Lea, MN 56007), were planted ~5.5 cm below the soil surface in 3.8 cm diameter x 21 cm depth, 164 mL conical plastic pots, one seed per pot.  Each pot contained a soil mix composed of 50% Sungro Premium Grade Vermiculite, 40% Sungro Canadian Sphagnum Peat Moss, and 10% Coarse Krum Perlite dampened with 30 ml of tap water.  Plants were watered twice weekly and allowed to grow at room temperature (19-20 C) with ambient lighting until 17 days old at which point viral inoculations occurred.  The plants were then moved to growth chambers (Percival Scientific) under 25C, 18-hour light, 6-hour dark cycles until 37 days old, at which point they were destructively harvested.  The plants were randomized spatially for host species, aphid vector, and nutrient treatment.

**Virus detection**

Virus detection methods reflect those reported in (Shoemaker et al. 2019). Total RNA extraction was carried out using a modified version of TRIzol® Reagent RNA-extraction protocol (Invitrogen^TM^).  Between 50 and 100 mg of each sample tissue was weighed, recorded, and cut into 1-2 mm pieces using scissors and placed into a 2 ml plastic screw cap microcentrifuge tube containing one 4.5 mm cal. steel BB (Copperhead). After adding 500 ul of TRIzol® Reagent, the tissue was ground in a Mini-Beadbeater-16 (Biospec Products) at 10 second intervals until solution was fully homogenized.  After a 4-minute incubation period at room temperature, 100 ul of chloroform was added to each tube and shaken for 15 seconds by hand.  Each sample was incubated for 3 minutes at room temperature, followed by cold centrifugation (4C) at 7,000g for 15 minutes.  The aqueous phase was then transferred to a new microcentrifuge tube and 100 ul of isopropanol was added to each sample. Samples were shaken by hand then incubated at room temperature for 10 minutes.  Samples were cold centrifuged at 7,000g for 10 minutes. The supernatant was discarded, and 1 ml of 75% ethanol was added. Samples were briefly vortexed and cold centrifuged again at 7,000g for 5 minutes.  The supernatant was discarded and the pellet containing RNA dried in the tube for a minimum of 30 minutes.  The pelleted RNA was then dissolved in 27.5 ul of RNase-free water and allowed to incubate for 10 minutes at 58C.  RNA quantification was performed using a NanoDrop^TM^ 2000c Spectrophotometer (Thermo Fisher Scientific).  Following quantification, the samples were placed on ice for at least 5 minutes and then stored at -80C.

Reverse transcription polymerase chain reaction (RT-PCR) was performed to generate complementary DNA (cDNA). For each sample, a mixture of 0.5 ul of random hexamers (1ug/ul) and 4.5 ul of RNA were quick spun and then heated in a S1000^TM^ Thermal Cycler (Bio-Rad) at 70C for 5 minutes. RT-PCR reactions were carried out in 20 ul reactions with 4 ul of 5x Reaction Buffer (ImProm-II^TM^ Reverse Transcriptase (Promega)), 1.2 ul of MgCl_2_ (25mM), 1 ul of dNTPs (10mM), 0.5 ul of Recombinant *RNasin*® Ribonuclease Inhibitor (Promega) (40U/ul), 1 ul of ImProm-II^TM^ Reverse Transcriptase (Promega), 7.3 ul of RNase free water, 0.034 ul of T4 Gene 32 Protein (New England BioLabs), and the 5 ul of random hexamer/RNA mixture. RT-PCR reaction tubes were briefly vortexed, spun, and placed in a thermal cycler with the following conditions: 5 min at 25C, 60 min at 45C, and finally 15 min at 70C. The cDNA was stored at -20C.

The presence of BYDV-PAV in plant tissue was determined using polymerase chain reaction (PCR) with the cDNA described above. Two BYDV-PAV primers, which are specific to the P3 coat protein gene - a conserved region that reduces the chance of false negatives - were used to isolate and amplify a 206 base pair region. PCR was carried out in 20 ul reactions with 2 ul of 10x buffer, 2.8 ul MgCl_2_ (25mM), 10.4 ul nanopure water, 0.8 ul of each forward and reverse primer (10 uM), 0.8 ul dNTPs (10 mM), 0.4 ul HotStarTaq® DNA Polymerase (Qiagen), and 0.068 ul T4 Gene 32 Protein (New England BioLabs).  Using forward primer: 5' – CCT TAA AGC CAA CTC TTC CG - 3' (PAV_3082_F), and reverse primer: 5' - TAG CTA GCC AGG GCT GAT T - 3' (PAV_3288_R), the target region was amplified in a thermal cycler using the following procedure: initial heating to 95C for 15 min; a step-down segment of 95C for 30 sec, 59C for 30 sec, and 72C for 1 min (reducing from 59C to 54C in 1C increments); 29 cycles of 95C (30 sec), 54C (1 min), and 72C (1 min); and 72C for 10 min. The amplified target DNA was loaded into a 2.0% UltraPure Agarose-1000 (Thermo Fisher Scientific) gel mixed with SYBR Safe DNA Gel Stain (Invitrogen^TM^) and run at 125 V for approximately 30 minutes alongside a GeneRuler 100bp DNA Ladder (Thermo Fisher Scientific). DNA was visualized using Gel Doc^TM^ EZ Imager (Bio Rad).

**Sensitivity Analysis**

We conducted a sensitivity analysis to examine the relative importance of the observed variation in transmission from plants to vectors ($\beta_{1}$) relative to the other demographic and transmission parameters. To do so, we simulated our model 10,000 times, exploring multi-dimensional parameter space by allowing multiple parameters to vary simultaneously. We drew each value for parameters $r_{h}$, $r_{i}$, $K$, $\beta_{1}$, $\beta_{2}$, $\mu$, and $\alpha$ from uniform distributions of their observed ranges (Supplementary Table D1). We defined observed ranges for each parameter as the 95% confidence interval reported in the literature for $r_{h}$, $r_{i}$, $\beta_{2}$, $\mu$, and $\alpha$ (Dixon and Glen, 1971; Ward *et al.*, 1998; Jiménez-Martínez and Bosque-Pérez, 2004; Jiménez-Martínez *et al.*, 2004), where the range for $\alpha$ is the full range of observed for both infected and healthy aphids, as calculated following Shaw et al. (2017). We varied *K* by plus or minus 50% of its value, as we could not find estimates of its confidence interval from the literature. We allowed $\beta_{1}$ to vary across the range of observed values from our experiments. We then applied multiple linear regression to determine the combined effects of all parameters on the rate of disease spread, as defined by the number of days it takes to reach 50% infection of hosts. We standardized all predictors (i.e. parameters) in the multiple regression to have a mean of zero and standard deviation of 1, so that all regression coefficients represent effect sizes.

From our sensitivity analysis, we see that our observed variation in transmission from vectors to plants has a strong impact on disease spread, where increased transmission decreases the number of days it takes to reach 50% infection. This was the strongest effect of all model parameters. Vector departure from host plants ($\alpha$) had a strong positive effect, where greater dispersal decreased disease spread due to the high mortality rate of dispersing aphids. Similarly, increasing mortality ($\mu$) had a slight positive effect, while increasing vector growth rates ($r_{h}$, $r_{i}$), carrying capacity ($K$), and plant to vector transmission ( $\beta_{2}$) decreased the days to 50% infections, but had a relatively small effect size.

**^References:^**

Bonett, D. G. and Price, R. M. (2012) ‘Adjusted Wald Confidence Interval for a Difference of Binomial Proportions Based on Paired Data’, *Journal of Educational and Behavioral Statistics*, 37(4), pp. 479–488. doi: 10.3102/1076998611411915.

Dixon, A. F. G. and Glen, D. M. (1971) ‘Morph determination in the bird cherry‐oat aphid, Rhopalosiphum padi L’, *Annals of Applied Biology*, 68(1), pp. 11–21. doi: 10.1111/j.1744-7348.1971.tb04633.x.

Jiménez-Martínez, E. S. and Bosque-Pérez, N. A. (2004) ‘Variation in Barley Yellow Dwarf Virus Transmission Efficiency by Rhopalosiphum padi (Homoptera: Aphididae) after Acquisition from Transgenic and Nontransformed Wheat Genotypes’, *Journal of Economic Entomology*, 97(6), pp. 1790–1796. doi: 10.1603/0022-0493-97.6.1790.

| **Supplementary Table A1.** Transmission coefficients $\beta_{1}$ for each modeled scenario | | | | | | | | | |  |  | |  |  |
| --- | --- | --- | --- | --- | --- | --- | --- | --- | --- | --- | --- | --- | --- | --- |
|  | |  |  |  |  | | |  | |  |  | |  | |
| Factor | Treatment | | Round | $\boldsymbol{\beta}_{\boldsymbol{1}}*$ | | | 95% CI* | | Days to 50% infection† | | | Figure, Panel | |  |
| **Vector** | *S. avenae* | | 1 | 0.0538 | | 0.0449—0.0638 | | | 68 | | | S1, A | |  |
|  | *R. padi* | | 1 | 0.0688 | | 0.0578—0.0843 | | | 63 | | | S1, A | |  |
|  | *S. avenae* | | 2 | 0.0594 | | 0.0471—0.0756 | | | 66 | | | S1, B | |  |
|  | *R. padi* | | 2 | 0.0891 | | 0.0707—0.1178 | | | 59 | | | S1, B | |  |
| **Host** | *A. sativa* | | 1 | 0.0653 | | 0.0544—0.0780 | | | 64 | | | S1, C | |  |
|  | *H. vulgare* | | 1 | 0.0564 | | 0.0461—0.0682 | | | 67 | | | S1, C | |  |
|  | *A. sativa* | | 2 | 0.0919 | | 0.0731—0.1190 | | | 58 | | | S1, D | |  |
|  | *H. vulgare* | | 2 | 0.0577 | | 0.0445—0.0713 | | | 66 | | | S1, D | |  |
| **Nutrient** | Control | | 1 | 0.0901 | | 0.0710—0.1158 | | | 59 | | | 2, A | |  |
|  | Phosphorus | | 1 | 0.0481 | | 0.0364—0.0635 | | | 70 | | | 2, A | |  |
|  | Control | | 2 | 0.0764 | | 0.0559—0.1039 | | | 61 | | | 2, B | |  |
|  | Phosphorus | | 2 | 0.0886 | | 0.0636—0.1297 | | | 59 | | | 2, B | |  |

* $\beta_{1}$ and 95% confidence intervals derived from the experiment

† Days to 50% infection derived from the dynamical simulations

**Supplementary Table B1.** BYDV infection prevalence. Lower and upper confidence intervals, and margin of error calculated using Adjusted Wald method (Bonett and Price, 2012).

| Treatment, Round | Proportion of infected plants per total plants | Lower confidence interval | Upper confidence interval | Margin of error |
| --- | --- | --- | --- | --- |
| *R. padi,* Round 1 | 0.747 | 0.678 | 0.806 | 0.064 |
| *R. padi,* Round 2 | 0.767 | 0.688 | 0.831 | 0.072 |
| *S. avenae,* Round 1 | 0.656 | 0.584 | 0.721 | 0.069 |
| *S. avenae,* Round 2 | 0.695 | 0.607 | 0.771 | 0.082 |
| *H. vulgare,* Round 1 | 0.672 | 0.600 | 0.737 | 0.069 |
| *H. vulgare*, Round 2 | 0.664 | 0.575 | 0.743 | 0.084 |
| *A. sativa,* Round 1 | 0.729 | 0.660 | 0.789 | 0.064 |
| *A. sativa,* Round 2 | 0.796 | 0.718 | 0.856 | 0.069 |
| P addition, Round 1 | 0.618 | 0.514 | 0.712 | 0.099 |
| P addition, Round 2 | 0.782 | 0.655 | 0.872 | 0.109 |
| N addition, Round 1 | 0.710 | 0.610 | 0.793 | 0.091 |
| N addition, Round 2 | 0.742 | 0.625 | 0.833 | 0.104 |
| NP addition, Round 1 | 0.643 | 0.536 | 0.737 | 0.101 |
| NP addition, Round 2 | 0.667 | 0.533 | 0.778 | 0.123 |
| Control no nutrient, Round 1 | 0.835 | 0.745 | 0.899 | 0.077 |
| Control no nutrient, Round 2 | 0.737 | 0.628 | 0.823 | 0.098 |

**Supplementary Table C1. Logistic regression model selection using dredge function**

| **cond((int))** | **disp((int))** | **cond(host)** | **cond(round)** | **cond(n)** | **cond(p)** | **cond(vec)** | **cond(host:round)** | **cond(host:n)** | **cond(host:p)** | **cond(host:vec)** | **cond(round:n)** | **cond(round:p)** | **cond(round:vec)** | **cond(n:p)** | **cond(n:vec)** | **cond(p:vec)** | **cond(host:round:n)** | **cond(host:round:p)** | **cond(host:round:vec)** | **cond(host:n:p)** | **cond(host:n:vec)** | **cond(host:p:vec)** | **cond(round:n:p)** | **cond(round:n:vec)** | **cond(round:p:vec)** | **cond(N:P:vec)** | **df** | **logLik** | **AICc** | **delta** | **weight** |
| --- | --- | --- | --- | --- | --- | --- | --- | --- | --- | --- | --- | --- | --- | --- | --- | --- | --- | --- | --- | --- | --- | --- | --- | --- | --- | --- | --- | --- | --- | --- | --- |
| 2.122 | + | + | 0.553 | -1.695 | -2.153 | + | + | + | + | NA | 1.214 | 0.748 | NA | 1.729 | NA | + | NA | + | NA | + | NA | NA | -1.977 | NA | NA | NA | 17 | -261.19 | 557.421 | 0 | 0.027 |
| 2.36 | + | + | 0.528 | -1.735 | -2.16 | + | + | + | + | + | 1.246 | 0.716 | NA | 1.752 | NA | + | NA | + | NA | + | NA | NA | -2.01 | NA | NA | NA | 18 | -260.2 | 557.564 | 0.143 | 0.025 |
| 1.273 | + | + | 1.147 | -0.012 | -0.803 | + | + | + | + | NA | NA | -0.221 | NA | -0.965 | NA | + | NA | + | NA | + | NA | NA | NA | NA | NA | NA | 15 | -263.48 | 557.764 | 0.344 | 0.023 |
| 1.48 | + | + | 1.138 | -0.01 | -0.785 | + | + | + | + | + | NA | -0.269 | NA | -0.986 | NA | + | NA | + | NA | + | NA | NA | NA | NA | NA | NA | 16 | -262.56 | 558.032 | 0.611 | 0.02 |
| 1.853 | + | + | 0.56 | -1.683 | -1.621 | + | + | + | + | NA | 1.202 | 0.69 | NA | 1.734 | NA | NA | NA | + | NA | + | NA | NA | -1.993 | NA | NA | NA | 16 | -263.02 | 558.952 | 1.531 | 0.012 |
| 2.261 | + | + | 0.527 | -1.937 | -2.206 | + | + | + | + | NA | 1.245 | 0.766 | NA | 1.795 | + | + | NA | + | NA | + | NA | NA | -1.986 | NA | NA | NA | 18 | -260.92 | 559.01 | 1.589 | 0.012 |
| 2.062 | + | + | 0.543 | -1.719 | -1.598 | + | + | + | + | + | 1.231 | 0.658 | NA | 1.752 | NA | NA | NA | + | NA | + | NA | NA | -2.027 | NA | NA | NA | 17 | -262.04 | 559.12 | 1.697 | 0.011 |
| 2.336 | + | + | 0.41 | -2.114 | -2.166 | + | + | + | + | NA | 1.516 | 0.743 | NA | 1.763 | NA | + | + | + | NA | + | NA | NA | -1.989 | NA | NA | NA | 18 | -261 | 559.157 | 1.736 | 0.011 |
| 2.504 | + | + | 0.503 | -1.982 | -2.218 | + | + | + | + | + | 1.272 | 0.736 | NA | 1.818 | + | + | NA | + | NA | + | NA | NA | -2.013 | NA | NA | NA | 19 | -259.94 | 559.162 | 1.741 | 0.011 |
| 2.58 | + | + | 0.381 | -2.162 | -2.177 | + | + | + | + | + | 1.554 | 0.713 | NA | 1.793 | NA | + | + | + | NA | + | NA | NA | -2.027 | NA | NA | NA | 19 | -260.01 | 559.308 | 1.887 | 0.01 |

**Supplementary Table D1. Parameters, description, and their values for disease transmission model (eq. 3 – eq. 6).** Mean values are used for Fig. 2 and Appendix 1, Fig. A1. Model parameters are drawn uniformly at random from the range listed in Appendix 1, Fig B1.

| Parameter | Description | Mean | Range |
| --- | --- | --- | --- |
| $r_{h}$ | Intrinsic growth rate of vectors on healthy hosts | 0.186 | 0.184-0.188 |
| $r_{i}$ | Intrinsic growth rate of vectors on infected hosts | 0.263 | 0.247-0.279 |
| $K$ | Carrying capacity of vectors on a single host | 100 | 50-100 |
| $\beta_{1}$ | Transmission coefficient from vector to plant host | Varies across treatments | 0.0364-0.1297 |
| $\beta_{2}$ | Transmission coefficient from plant host to vector | 0.68 | 0.52-0.74 |
| $\mu$ | Dispersal-induced vector mortality rate | 0.994 | 0.983-0.998 |
| $\alpha$ | Vector departure rate from hosts | 0.1353 | 0.122-0.143 |


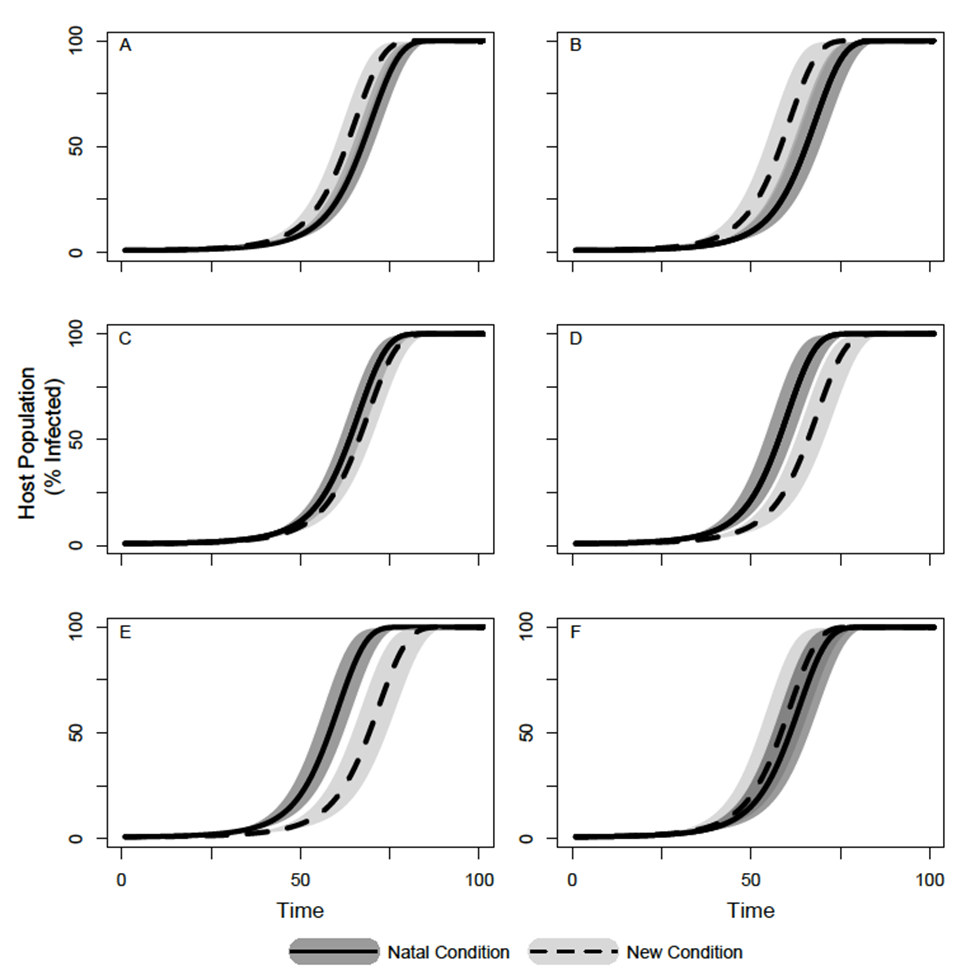

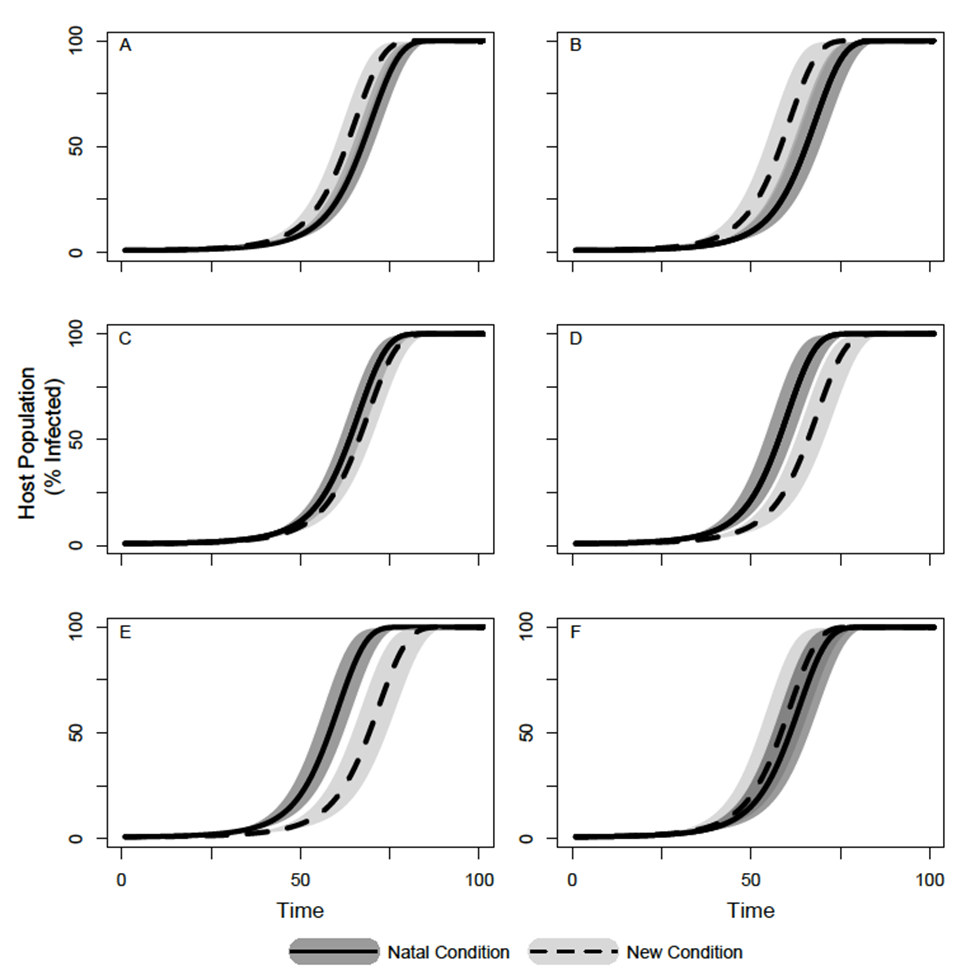


**Host Population (% Infected)**

**Natal condition**

**New condition**

(d)

(c)

(b)

(a)

**Time**

**Time**

**Round 2**

**Round 1**

**Supplementary material Appendix 1, Fig. A1**. Estimated virus spread in hosts comparing natal (solid lines) and new (dashed lines) conditions across Round 1 (first column) and Round 2 (second column). We show comparisons for changing from the natal *S. avenae* to *R. padi* vector (panels a and b) and *A. sativa* to *H. vulgare* host (panels c and d). Shaded regions show 95% confidence intervals, estimated from the bootstrapped 95% confidence intervals of vector to plant transmission coefficients ($\beta_{1})$. Models were parameterized such that $\beta_{1}$ was estimated directly from experiments, $\beta_{2}=0.68$ (Jiménez-Martínez and Bosque-Pérez, 2004), $r_{h}=0.186$, $r_{i}=0.263$ (Jiménez-Martínez *et al.*, 2004), $K=100$, $\alpha=0.1353$(Dixon and Glen, 1971; Jiménez-Martínez *et al.*, 2004), and $\mu=0.994$(Ward *et al.*, 1998).


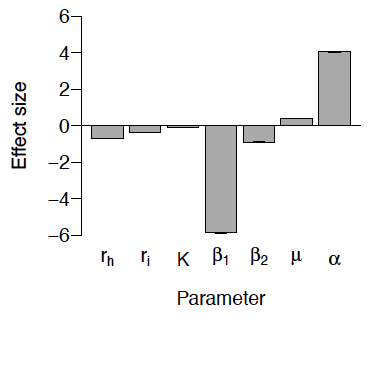


**Supplementary material Appendix 1, Fig. B1.** Effect size of variation in model parameters on the number of days it takes to reach 50% infection of the host population. Error bars show 1 standard error.
